# Single-Molecule Force Spectroscopy on the N2A Element of Titin: Effects of Phosphorylation and CARP

**DOI:** 10.3389/fphys.2020.00173

**Published:** 2020-03-18

**Authors:** Thomas Lanzicher, Tiankun Zhou, Chandra Saripalli, Vic Keschrumrus, John E. Smith III, Olga Mayans, Orfeo Sbaizero, Henk Granzier

**Affiliations:** ^1^Department of Cellular & Molecular Medicine, The University of Arizona, Tucson, AZ, United States; ^2^Department of Engineering and Architecture, University of Trieste, Trieste, Italy; ^3^Department of Biology, University of Konstanz, Konstanz, Germany

**Keywords:** titin, passive stiffness, spring elements, post-translational modification, mechano-signaling

## Abstract

Titin is a large filamentous protein that forms a sarcomeric myofilament with a molecular spring region that develops force in stretched sarcomeres. The molecular spring has a complex make-up that includes the N2A element. This element largely consists of a 104-residue unique sequence (N2A-Us) flanked by immunoglobulin domains (I80 and I81). The N2A element is of interest because it assembles a signalosome with CARP (Cardiac Ankyrin Repeat Protein) as an important component; CARP both interacts with the N2A-Us and I81 and is highly upregulated in response to mechanical stress. The mechanical properties of the N2A element were studied using single-molecule force spectroscopy, including how these properties are affected by CARP and phosphorylation. Three protein constructs were made that consisted of 0, 1, or 2 N2A-Us elements with flanking I80 and I81 domains and with specific handles at their ends for study by atomic force microscopy (AFM). The N2A-Us behaved as an entropic spring with a persistence length (Lp) of ∼0.35 nm and contour length (Lc) of ∼39 nm. CARP increased the Lp of the N2A-Us and the unfolding force of the Ig domains; force clamp experiments showed that CARP reduced the Ig domain unfolding kinetics. These findings suggest that CARP might function as a molecular chaperone that protects I81 from unfolding when mechanical stress is high. The N2A-Us was found to be a PKA substrate, and phosphorylation was blocked by CARP. Mass spectrometry revealed a PKA phosphosite (Ser-9895 in NP_001254479.2) located at the border between the N2A-Us and I81. AFM studies showed that phosphorylation affected neither the Lp of the N2A-Us nor the Ig domain unfolding force (F_unfold_). Simulating the force-sarcomere length relation of a single titin molecule containing all spring elements showed that the compliance of the N2A-Us only slightly reduces passive force (1.4%) with an additional small reduction by CARP (0.3%). Thus, it is improbable that the compliance of the N2A element has a mechanical function *per se*. Instead, it is likely that this compliance has local effects on binding of signaling molecules and that it contributes thereby to strain- and phosphorylation- dependent mechano-signaling.

## Introduction

Titin is a giant protein located in the sarcomere of striated muscle, where it plays critical roles in muscle health and disease (Granzier and Labeit, 2005; LeWinter and Granzier, 2014; Linke, 2018). The spring segment of titin, located in the I-band region of the molecule, has been extensively studied (Trombitas et al., 1995; Granzier et al., 1996). In response to sarcomere stretch, titin’s molecular spring generates passive force, which is important for maintaining the structural integrity of the contracting sarcomere, limiting sarcomere length inhomogeneity along myofibrils, and tuning the level of active force during contraction (Horowits et al., 1989; Labeit and Kolmerer, 1995; Fukuda et al., 2008; Anderson and Granzier, 2012). The molecular spring segment of titin consists of multiple extensible elements that each behave as entropic wormlike chains (WLCs) but with distinct contour length (Lc) and persistence length (Lp) (Linke and Granzier, 1998; Li et al., 2002; Watanabe et al., 2002a, b). Two of these spring elements are found in all muscle types: the tandem Ig segments (serially linked immunoglobulin domains organized in proximal, middle, and distal segments) and the PEVK segment (Freiburg et al., 2000). A third spring element is the cardiac-specific N2B element that contains a large unique sequence (∼550 amino acids) that provides ∼200 nm extensibility to cardiac titin (Helmes et al., 1999). A fourth element, located between the middle tandem Ig segment and the PEVK segment, is the N2A element, found in skeletal muscle titin, fetal cardiac titin, and adult cardiac N2BA titin (Cazorla et al., 2000; Bang et al., 2001). The N2A element is the least well-studied portion of titin’s molecular spring. It contains four Ig domains and unique sequences, of which the 104-residue unique sequence (N2A-Us) with flanking Ig domains (I80 and I81) is a major component (Labeit and Kolmerer, 1995). The importance of the N2A element to muscle health is supported by a mouse model with a spontaneous mutation resulting in an in-frame deletion of part of the N2A element (the MDM model) that develops severe myopathy with early death (Garvey et al., 2002). Additionally, missense mutations in the N2A-Us have been linked to cardiomyopathy (Arimura et al., 2009).

N2A-Us had been assumed to be largely unstructured, but recent evidence suggests that it contains α-helical secondary structure (Zhou et al., 2016; Tiffany et al., 2017). It is often assumed that the N2A-Us contributes significantly to the extensibility of titin, but this remains to be established. Here we studied the mechanical properties of the N2A-Us and its flanking Ig domains using single-molecule force spectroscopy. Protein constructs were engineered consisting of 0, 1, or 2 N2A-Us domains, flanked by I80 and I81, and with specific handles at the ends (a halo-tag at the C-terminus and a double cysteine at the N-terminus) for attachment in an atomic force microscope (AFM). We measured the biophysical characteristics of the N2A-Us and used these in a serially linked WLC model that incorporates all spring elements to simulate the role of the N2A-Us in the passive force-sarcomere length relation of the whole titin molecule.

The N2A element is also a protein-binding hub, as it interacts with calpains (calcium-dependent cysteine proteases) (Ojima et al., 2007; Hayashi et al., 2008; Lostal et al., 2019), smyd2 (Donlin et al., 2012; Voelkel et al., 2013; Munkanatta Godage et al., 2018), F-actin (Dutta et al., 2018), and a family of muscle-specific ankyrin repeat proteins, the MARPs (Miller et al., 2003). The MARP family member CARP (Cardiac Ankyrin Repeat Protein) is particularity interesting considering, for example, the importance of CARP-regulated Nkx-2.5-dependent signaling pathways (Witt et al., 2004), that CARP interacts with the signaling molecule myopalladin (Bang et al., 2001), and that mutations in CARP that increase its binding affinity to titin have been linked to cardiomyopathy (Arimura et al., 2009). Furthermore, in response to mechanical stress imposed on the heart, CARP is up-regulated and localizes to titin’s I-band region, where it co-localizes with the N2A element (Witt et al., 2004). In skeletal muscle, CARP is normally present at very low levels, but also, in this muscle type, CARP has been found to be highly upregulated under conditions of mechanical stress (Barash et al., 2004). A prime example is the unilateral diaphragm denervation model, where CARP protein levels are increased ∼400-fold in the denervated hemi-diaphragm (van der Pijl et al., 2018, 2019). It was recently shown that CARP interacts with both the N2A-Us and I81 (Zhou et al., 2016), and since this might affect the extensibility of the N2A-Us, we studied the effect of CARP on the mechanical properties of the N2A-Us and its flanking Ig domains.

It has been well-established that the mechanical properties of titin’s N2B and PEVK spring elements can be tuned via phosphorylation by kinases important in multiple cell signaling processes in striated muscle (e.g., ERK2, PKA, and PKG) (Fukuda et al., 2005; Kruger et al., 2009; Hidalgo and Granzier, 2013; Perkin et al., 2015). However, whether the N2A element can be phosphorylated and how this affects extensibility are less clear. Recent studies showed that PKA phosphorylates the N2A-Us (Lun et al., 2014; Adams et al., 2019), and earlier studies showed that the N2A-Us can be phosphorylated by PKG but not PKA (Kruger et al., 2009; Hisman et al., 2011). Hence, we also focused on N2A phosphorylation and how it affects the extensibility of N2A-Us and Ig domain unfolding.

## Materials and Methods

### Protein Engineering

**FIGURE 1:**
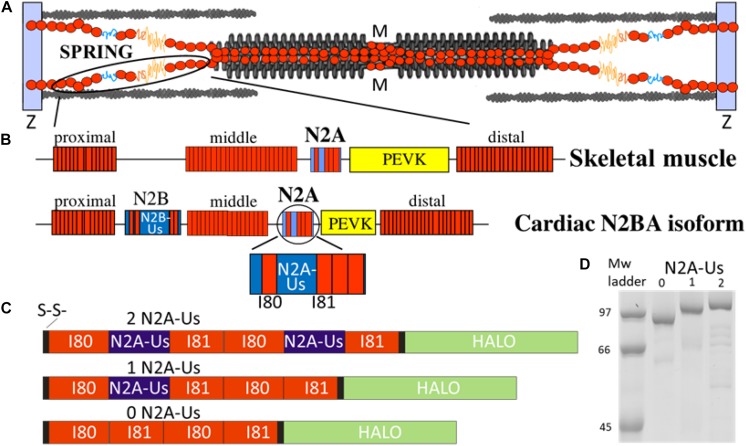
The N2A element is part of titin’s molecular spring. **(A)** Titin spans from the Z-disk to the M-band of the sarcomere; the I-band region of the molecule functions as a molecular spring. **(B)** Domain composition of the molecular spring region of skeletal muscle titin (top) and adult cardiac N2BA isoform (bottom) with expanded N2A element shown at bottom. Red rectangle: immuno-globulin domains making up a proximal, middle, and distal tandem Ig segment; blue: unique sequence; yellow: PEVK. **(C)** Domain composition of the three N2A constructs used in this study: all contain two I80 and two I81 immunoglobulin domains (red) and either 0, 1, or 2 N2A-Us elements (blue). **(D)** Expressed constructs are of the expected molecular weight with minimal signs of protein degradation.

Three different N2A recombinant protein constructs were made. The first consisted of the four Ig domains (I80, I81, I80, and I81), the second contained a single N2A-Us embedded between the first I80-I81 pair, and the third contained two N2A-Us elements, each embedded between a I80-I81 pair; these are referred to as the 0, 1, and 2 N2A-Us constructs, respectively (Figure 1). All constructs also contained a HaloTag at the C-terminus (for covalent attachment to a glass surface, via an ester bond with a chloroalkane ligand; see below) and a double cysteine at the N-terminus for attachment to a gold-coated AFM cantilever. The bacterial codon-optimized expression construct (and its derivatives) were synthesized by DNA2.0 (now Atum) and cloned into the pJ404 expression vector with ampicillin resistance, a lacO-flanked T5 promoter, and a strong ribosome binding site. The full-length expressed protein consists of Cys(x2)-His(x6)-[amino acids 9689-9988 of human Titin (NP_001254479.2)](x2)-TEV cleavage site-[amino acids 3-296 of HALO7 (AQS79242.1)]; see Supplementary Figures S1A,B. The derivative expression constructs have internal deletions of either the second or both unique sequence regions between Ig domains I80 and I81 (amino acids 9792-9895 of human Titin). Protein was expressed in BLR(DE3)pLysS cells (Novagen) by induction with 1 mM IPTG in LB overnight at 15C. Lysate was prepared from bacterial pellet using Lysis Buffer [20 mM Tris, 10 mM imidazole, 150 mM NaCl, 10% glycerol, 0.2% NP-40, 2 mM β-mercaptoethanol, complete protease inhibitor (Roche), pH 8] to disrupt bacterial cells with sonication pulses (using a Branson Sonifier). Proteins were purified using the His tag with batch Ni^2+^-NTA (Qiagen) affinity chromatography. Proteins were eluted with 330 mM imidazole in Lysis Buffer without detergent and exchanged into 10 mM HEPES, 150 mM NaCl, 1 mM EDTA, 10% glycerol, pH 7.2. Aliquots of the protein were frozen in liquid nitrogen and stored at −80°C until used.

The CARP experiments used a recombinant N-terminally truncated CARP construct corresponding to the titin-binding region of CARP, CARP^106–319^. This CARP construct contains all Ankyrin Repeat (AR) domains and has been shown to bind to the N2A-Us and I81 (Zhou et al., 2016). Binding of CARP to the N2A-Us and N2A-Us-I81 titin segments occurs upon mixing of the samples, as shown previously by size exclusion chromatography (SEC) and SEC-coupled to multi-angle laser light scattering (SEC-MALLS) (Zhou et al., 2016). Binding in this work was achieved similarly. The production of CARP has been described previously (Zhou et al., 2016). Briefly, human CARP^106–319^ (UniProtKB Q15327) was cloned into the pET-Trx1a vector. Proteins were expressed in Rosetta(DE3) *E. coli* (Novagen), and cells were harvested and lyzed in 25 mM HEPES pH7.5, 300 mm NaCl. The purification from supernatants followed Ni^2+^-NTA affinity chromatography, tag removal by TEV protease digestion, reverse affinity chromatography, and size exclusion chromatography [for additional details, see (Zhou et al., 2016)].

### Protein Phosphorylation

The purified recombinant proteins (containing 0, 1, or 2 N2A unique sequence regions) were used in phosphorylation assays along with no-kinase and kinase-plus-inhibitor controls by incubation in kinase buffer [final concentration in mM: NaATP 2.36, HEPES 6, imidazole 16, NaCl 90, K(C_2_H_5_COO) 28, Mg(CH_3_COO)_2_ 2.56, creatine phosphate 4, EGTA 4, EDTA 0.6, NaN_3_ 2, DTT 0.4, E-64 0.04, leupeptin 0.16, NaF 20, Na_3_VO_4_ 4] for 4 hr at 30°C along with the following. PKA phosphorylation: Incubated with 1.0 U/ul PKA catalytic subunit (Sigma-Aldrich P2645), ± 0.04 mM PKA inhibitor fragment 6-22 amide (Sigma-Aldrich P6062). PKG phosphorylation: Incubated with 1.2 mM cGMP (Sigma-Aldrich C5438), 25 U/ul PKG (Promega V5171), and 0.04 mM PKA inhibitor, ± inhibitor KT-5823 (Sigma-Aldrich K1388). ERK2 phosphorylation: Incubated with 1.3 U/ul activated recombinant human MAPK2 (Calbiochem 454854), ± ERK Inhibitor II/FR180204 (EMD Millipore 328007). Reactions were at 30°C for 4 h. Reactions were stopped by the addition of an equal volume of 2X reducing sample buffer (5% 2-mercaptoethanol, 60 mM Tris, 10% glycerol, 2% SDS, 0.005% bromophenol blue) and incubated at 95°C 10 min. Proteins were separated by 10% SDS-PAGE and visualized sequentially with Pro-Q Diamond phospho-stain (Invitrogen P33300) and either SYPRO Ruby Protein Stain (Invitrogen S12000) or Coomassie Blue stain for normalization of Pro-Q diamond signal. Scanned images were analyzed using One-D San (Scanalytics). Incubation with CARP: A 2:1 ratio (w/w) of CARP:N2A-Us recombinant protein was incubated for PKA phosphorylation as described above. Mass Spectrometry: PKA-phosphorylated recombinant protein (with two N2A-Us) and the matching no-kinase control were gel-purified to remove the kinase and other bacterial contaminants. TiO_2_ phosphopeptide enrichment (High-Select kit, Thermo Fisher Scientific A32993) was performed following the manufacturer’s recommendations, and samples were submitted to the University of Arizona Analytical and Biological Mass Spectrometry Core Facility. Tandem mass spectrometry (LC-MS/MS) of trypsin-digested samples used the LTQ Orbitrap Velos (Fisher Scientific), and MS spectra of peptides were analyzed with TurboSEQUEST. The Mass Spectrometry data for this study can be found at the Harvard Dataverse^1^. All other datasets for this study are included in the article/Supplementary Material.

### Attachment of Single-Molecules

The attachment chemistry was conducted according to Taniguchi and Kawakami (2010) and Popa et al. (2013). Briefly, glass slides were cleaned using piranha solution and silanized with (3-Aminopropyl)triethoxysilane. These amine-terminated surfaces were then reacted for 1 h with 10 mM NHS-PEG-Maleimide Cross-linker [SM(PEG), Thermo Fisher Scientific] dissolved in 50 mM Borax buffer, pH 8.5. After washing with double-distilled water, the surfaces were further reacted overnight with a 7.5 mM Thiol-PEG4-Chloroalkane ligand (HaloTag Thiol O4 ligand, Promega) dissolved in 50 mM Borax buffer pH 8.5. The reaction was quenched with 50 mM 2-mercaptoethanol. Just prior to an experiment, a protein aliquot was thawed, and the protein solution was added to the chloroalkane-containing glass surface for ∼60 min. For the CARP experiments, ∼6.5 μ CARP was then added to the slide, followed by incubation for 60 min. The surface was then washed with AFM buffer (10 mM HEPES pH 7.2, 150 mM NaCl, 1 mM EDTA), and AFM measurements were performed in the AFM buffer.

### Single-Molecule Force Spectroscopy

An MFP-3D AFM (Asylum Research, Santa Barbara, CA, United States) was used for force spectroscopy. Cantilevers were gold-coated, and their spring constant was determined at the beginning and end of each experiment through a thermal calibration procedure (typically ∼20 pN/nm) (Florin et al., 1995). The piezo motor moved the cantilever up and down and was set to operate at a pulling velocity of 400 nm/s. This speed makes comparisons to previous work possible, is a speed that falls within the physiological speed range (it corresponds to 2 × 400 nm/s per sarcomere or 1/3 lengths per second, assuming a sarcomere length of ∼2.4 μm), and, finally, it is a speed that allows single-molecule experiments to have a relatively high throughput. The cantilever tip was used to probe the glass surface and, by regularly moving the slide laterally by a short distance, different surface locations (and different molecules) were probed. The cantilever tip approached and retracted from the surface of the slide, and when an extension/retraction force curve was obtained, it was evaluated whether it displayed multiple regularly spaced (∼30 nm) saw-tooth force peaks (one for each Ig domain present), a larger force peak due to unfolding of the HaloTag, and a final peak due to detachment of the molecule from one of its attachment points. The obtained force-extension curves were analyzed with the wormlike chain (WLC) equation (Marko and Siggia, 1994):

(1) $$F=\frac{k_{B}\,T}{L_{p}}\,\left(\frac{1}{4\,{\left(1-\frac{z}{L_{c}}\right)}^{2}}-\frac{1}{4}+\frac{z}{L_{c}}\right)$$

F is the force, k_*B*_T is the thermal energy (k_*B*_ is Boltzmann’s constant, and T is the absolute temperature), Lp is the persistence length of the molecule, Lc the contour length, and z is the end-to-end length of the molecule. The persistence length, Lp, is the minimal distance along the backbone of the molecule over which tangent lines are correlated. As Lp increases, the molecule is less flexible, and lower force values are required to stretch the molecule to a given fractional extension.

Although the protein constructs have two sets of identical domains (two I80 and two I81 domains), the identical domains are expected to unfold sequentially and not simultaneously. Unfolding is a force-dependent and stochastic process where identical domains will rarely unfold at the same time. Instead, unfolding occurs one by one, with each unfolding event triggering a similar length gain. The classical AFM studies by Fernandez and colleagues on polyproteins that contain a large number of identical Ig domains (up to 12) show this clearly. See, for example (Carrion-Vazquez et al., 1999). Hence, identical domains in our protein constructs are expected to unfold sequentially.

Force clamp protocols were also performed to measure force-dependent unfolding. The cantilever was initially held onto the surface for 2 s to allow attachment to occur, and the cantilever was then moved away from the surface until the measured force matched a pre-determined set point, which initiated the force-clamp phase of the protocol. To control force, the proportional-integral-derivative feedback (PID feedback) with a 2-ms time resolution was used. Each time an unfolding event takes place, a sudden increase in the extension of the molecule is registered. The resulting length-versus-time traces (Oberhauser et al., 2001) exhibit staircases in which the height of each step serves as a fingerprint for the unfolding of a module, and time marks the unfolding dwell time, t, from the moment the force is applied. The force-dependence of the rate of unfolding has been shown to follow the Bell model (Bell, 2008).

(2) $$\alpha \,\left(F\right)=\alpha_{0}\,\exp\left(\frac{F\,\Delta \,x}{k_{B}\,T}\right)$$

where F is the pulling force, α_0_s the rate constant in the absence of external applied force, Δx is the distance to the transition state, and *k*_*B*_at the experimental room temperature is 4.114 pN⋅nm. This expression can be rearranged into:

(3) $$\ln\alpha \,\left(F\right)=\alpha_{0}+\frac{F\,\Delta \,x}{k_{B}\,T}$$

The model implies probabilistic behavior of the process with a single rate constant, which can be obtained from the single exponential fit to the average Ig domain failure trajectory, normalized by the length of the clamp events. The probability of unfolding over time, P(t), is given by:

(4) $$P\,\left(t\right)=1-e^{-\alpha \,t}$$

### Simulated Force-Sarcomere Length Relation of Single-Molecules

To determine how the compliance of N2A-Us affects the force-sarcomere length behavior of a single titin molecule in the sarcomere, the spring region of titin was simulated as four WLCs in series: (1) the tandem Ig segment (combined proximal, middle, and distal segments), (2) the PEVK segment, (3) the N2B-Uc, and (4) the N2A-Us. For a WLC, the external force is given by equation 1 (see above). Based on previous work (Trombitas et al., 1998a; Kellermayer et al., 2000, 2001, 2003; Trombitas et al., 2000; Li et al., 2001; Anderson and Granzier, 2012; Anderson et al., 2013; Chung et al., 2013), the contour lengths of tandem Ig segments, the N2B-Uc, and the PEVK were assumed to be 325 nm, 200 nm, and 350 nm, respectively, and their respective Lp values to be 10, 0.65, and 1.0 nm. Based on the present study (see below) the Lc of the N2A-Us was taken as 40 nm and the Lp as either zero (i.e., inextensible N2A-Us), 0.34 nm (no CARP), or 0.44 nm (CARP). Because spring elements are in series, they bear equivalent forces, and the fractional extension (z/L) at each force can be calculated. From the fractional extensions and the Lc value (above) the corresponding SLs were determined assuming that the inextensible Z-disk and A-band segments of titin are 700 nm long (per half sarcomere) (Trombitas et al., 1998b).

### Statistical Analysis

Statistical analysis was performed in GraphPad Prism (GraphPad Software, Inc.). A one-way ANOVA with a Bonferroni *post hoc* analysis was used to calculate adjusted *p*-values corrected for multiple comparisons. When non-normally distributed data were analyzed, a Kruskal–Wallis test was used. Normality was tested with the D’Agostino and Pearson test. A value of *p* < 0.05 was taken as significant. Values were also plotted in histograms that were fit with Gaussian curves. Results are shown as mean ± SEM.

## Results

The location of titin in the sarcomere and the domain composition of titin’s spring region are shown in Figures 1A,B. The best-characterized spring elements are the serially linked immunoglobulin domains, organized in proximal, middle, and distal segments, the N2B element (cardiac-specific) and the PEVK. The N2A element is found in both N2BA cardiac titin and skeletal muscle titin and contains a large unique sequence (N2A-Us, 104 residues), flanked at one end by I80 and at the other by I81 (Bang et al., 2001). We studied titin’s N2A element using single-molecule force spectroscopy. Three distinct constructs were used, all with two I80 and two I81 domains but with either 0, 1, or 2 N2A-Us elements (see Figures 1C,D). Additionally, all constructs contained a Halo-tag at one end and a double cysteine at the other (Figure 1C and Supplementary Figure S1) for specific attachment to a functionalized slide and gold-coated cantilever tip, respectively.

**FIGURE 2:**
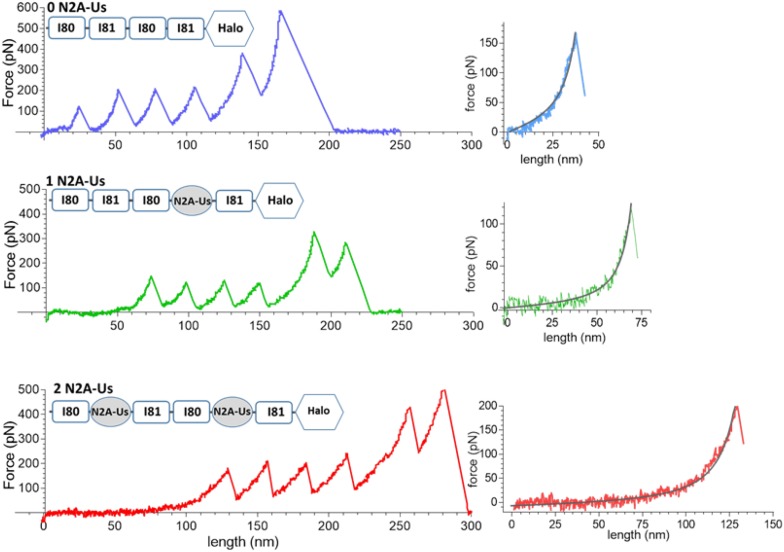
Example force-extension curves. Constructs that were pulled had 0, 1, and 2 N2A-Us; the domain composition of the constructs is shown at the top of each trace. **(Left)** Full traces. Each trace has a low-force extension phase that is followed by six force peaks, the first five of which are likely due to Ig domain and Halo-tag unfolding and the sixth due to the molecule breaking loose from one of its anchors. **(Right)** Initial force traces leading up to the first force peak shown on expanded scales with superimposed black WLC fits. The length of the initial low force varies with the number of N2A-Us elements.

Examples of force-extension curves for each of the constructs are depicted in Figure 2. There is an initial low-force phase, likely dominated by extension of the N2A-Us, followed by four force peaks at similar height, likely due to Ig domain unfolding. The fifth peak occurs at a higher force and is likely due to unfolding of the HaloTag. The final force peak represents the breaking loose of the molecule from one of its attachment points.

**FIGURE 3:**
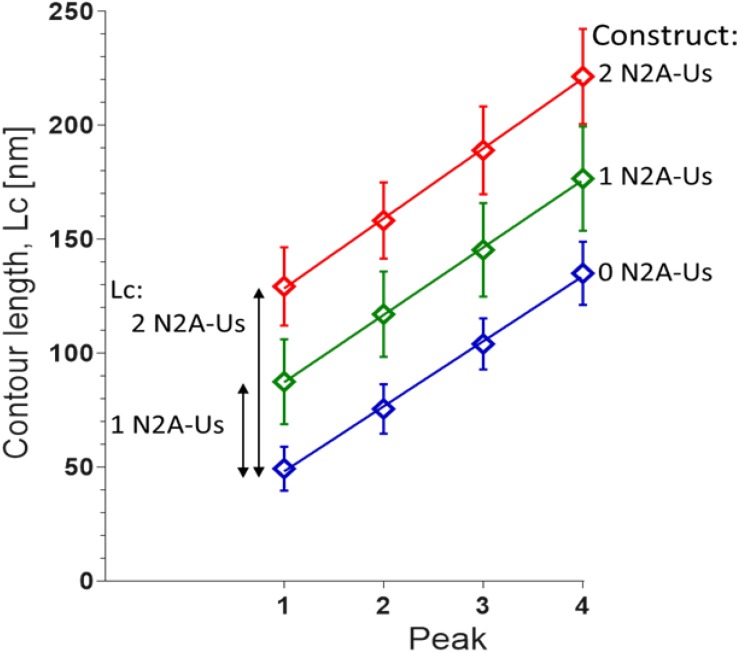
Contour length (Lc) of N2A constructs. Lc was measured by fitting the force-extension traces leading up to each of the first four unfolding peaks with the WLC equation. The slopes of the linear fits are 28.6 ± 0.8 nm, 29.6 ± 0.4 nm, and 30.7 ± 0.6 nm for 0, 1, and 2 N2A-Us-containing constructs, respectively. These values reflect the Lc gain due to Ig domain unfolding. The differences in the offsets of the linear regression fits reflect the Lc of 1 and 2 N2A-Us element, 38.1 and 78.2 nm, respectively.

The contour length (Lc) of the force-trace leading up to each of the force peaks was determined by fitting the data to the wormlike chain (WLC) force equation (Methods). Lc increased linearly with peak number with slopes that were similar in the three constructs (∼30 nm, Figure 3), as expected since the different constructs contain identical Ig domains. This ∼30 nm value is consistent with the notion that each force peak is derived from the length of an unfolded Ig domain (∼35 nm) minus the length of a folded domain (∼5 nm). The offset of the lines reflects the Lc of the N2A-Us (Figure 3), with the Lc of a single N2A-Us at ∼39 nm. The N2A-Us contains 104 residues, and the Lc value indicates an average residue spacing of 0.38 nm.

The fifth unfolding peak had a mean value of 306 ± 12 pN, significantly higher (*p* < 0.0001) than the 163 ± 3 pN of the preceding force peak (*n* = 293). This is consistent with the fifth peak being derived from the unfolding of the Halo tag and preceding peaks from unfolding of Ig domains. Thus, the available constructs make it possible to study the unfolding forces of the Ig domains (force peaks 1–4) and the characteristics of N2A-Us (low-force phase preceding the 1st unfolding peak). Furthermore, we used the constructs to study the effects of CARP on Ig domain unfolding and extension of the N2A-Us.

**FIGURE 4:**
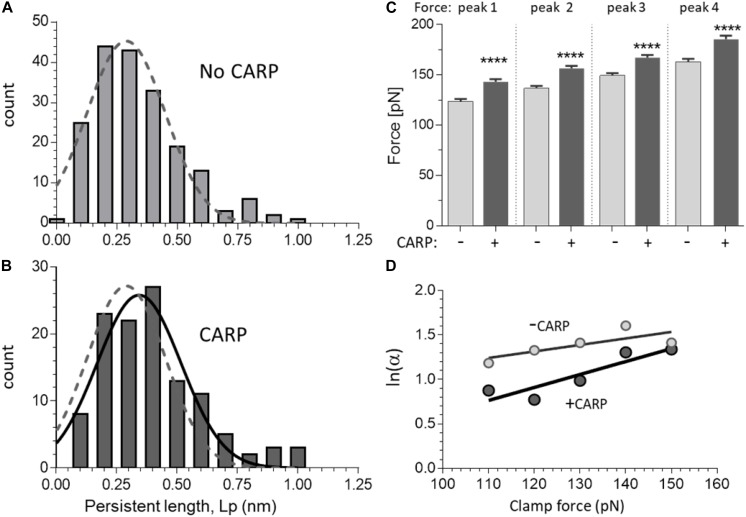
Effect of CARP on the force-extension curves of N2A constructs. A and B) Persistence length (Lp) distribution of N2A-Us in the absence of CARP **(A)** and in the presence of CARP **(B)**. Data obtained from constructs with 1 and with 2 N2A-Us elements were not statically different, which is expected, and were pooled. Gaussian fits are shown. The fit of the without-CARP data was overlaid in panel **(B)** as a dashed line. Note that the Lp graph is slightly right-shifted by CARP. **(C)** Peak force of peaks 1–4 measured with and without CARP. Force levels of all force peaks are higher in the presence of CARP. **(D)** Logarithm of unfolding rate α as a function of force, measured in force-clamp experiments. The two lines represent the fitting of the Bell expression (see section “Materials and Methods”) and give values of α_*o*_ = 1.55 s^–1^, Δx = 0.30 Å for the without-CARP data and α_*o*_ = 0.43 s^–1^, Δx = 0.60 Å for the CARP data. ****p < 0.0001.

The persistence length (Lp) of the N2A-Us was determined from the WLC fit to the low-force phase preceding the first unfolding peak. Both without and with CARP, the Lp distribution ranged from ∼0.1 to 1.0 nm (Figures 4A,B). CARP did not affect Lc (39 nm) but increased Lp from 0.34 ± 0.01 nm (*n* = 190) to 0.44 ± 0.03 nm (*n* = 56) (p: 0.002).

The Ig domain unfolding force (F_unfold_) increased with peak number, which is a common feature of multi-domain proteins (see section “Discussion”). The F_unfold_ varied from ∼125 to ∼165 pN, which indicates that these domains are amongst the least stable of the studied titin Ig domains (Watanabe et al., 2002a). The presence of CARP had no effect on Lc (determined from the slope of Lc vs. peak number), but CARP significantly increased unfolding force by an average of ∼20 pN (see Figure 4C).

The increased F_unfold_ in the presence of CARP suggests that the mechanical stability of the constructs is increased by CARP. To determine the effect of CARP on the unfolding kinetics, we performed force clamp experiments in which the molecule was held at a constant force and the resulting staircase-like increase in the length of the tethered molecule was determined. We performed load clamps in the 100–150 pN range, with mean clamp values in all experiments of 135.1 ± 0.9 pN and 132.7 ± 0.8 pN for the construct with and without CARP, and with mean step-size of 27.0 ± 0.1 nm without CARP (*n* = 630) and 26.8 ± 0.1 nm with CARP (*n* = 627). The rate of unfolding α was determined at different clamp forces from the probability of unfolding over time *P*(*t*) = 1−*e*^−α*t*^. The unfolding rate at each force level was higher without CARP than with CARP, e.g., at 100 pN, α was 3.2 s^–1^ and 2.4 s^–1^, respectively. Plotting ln(α) at a range of clamp forces results in a linear relationship of the form ln a = a_0_ + FΔx/(K_*B*_T); α_0_ is the unfolding rate constant in the absence of external applied force, Δx is the distance to the transition state, *k*_*B*_s the Boltzmann constant, and T is absolute temperature *k*_*B*_(T 4.11 pN⋅nm). The line fit to the results obtained without CARP was distinct from the one with CARP (Figure 4D), and the slopes and Y-intercepts of the linear regression fits revealed α_0_0.55 s^–1^ without CARP and 0.43 s^–1^ with CARP. These results indicate that CARP slowed the unfolding kinetics of the Ig domains that flank the N2A-Us, likely explaining the higher average unfolding force in the presence of CARP.

### Effect of Protein Kinase a (PKA) Phosphorylation

**FIGURE 5:**
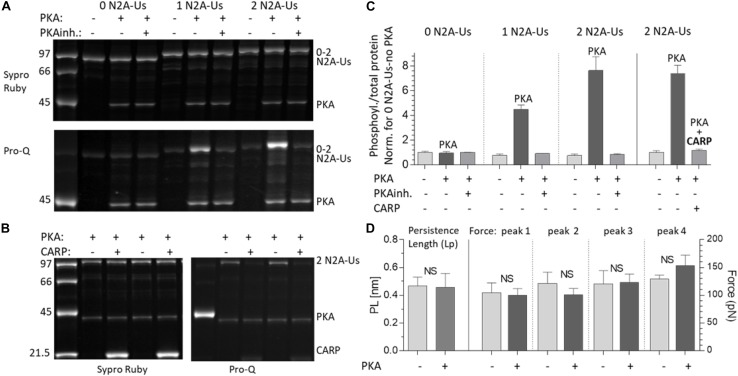
PKA phosphorylates the N2A-Us; phosphorylation is blocked by CARP and does not affect the molecular mechanics of the N2A element. **(A)** The N2A constructs with 0, 1, and 2 N2A-Us elements were incubated with PKA with and without PKA inhibitor (PKAinh.). Top: protein-stained gel (Sypro-ruby stain); bottom: Pro-Q Diamond phosphoprotein-stained gel (same gel). Constructs are phosphorylated by PKA but only when N2A-Us is present and in a manner that scales with the number of N2A-Us elements contained in the construct. This is visually apparent **(A)** and is clear from the gel analysis (**C**, left three triplicate sets of bars). **(B)** PKA phosphorylation of the N2A construct (2 N2BA-Uc elements) with and without CARP. Left: protein-stained gel (Sypro-Ruby stain). Right: Pro-Q Diamond phosphoprotein-stained gel. It is visually apparent **(B)** and is clear from the analysis (**C**, right three bars) that CARP blocks N2A-Us phosphorylation. Note on the pro-Q Diamond-stained gel that PKA is auto-phosphorylated and that this is unaffected by CARP. **(D)** Lp of the N2A-Us (left pair of bars and left *Y*-axis) and Ig F_unfold_ (right four bar pairs and right *Y*-axis) with and without PKA phosphorylation. Phosphorylation does not affect the Lp of the N2A-Us or the Ig domain F_unfold_.

Various kinases are known to phosphorylate the N2B and PEVK spring elements and modulate titin’s stiffness (Hidalgo and Granzier, 2013). However, whether the N2A element can be phosphorylated and the resultant effect on stiffness are less clear. Using the 3 N2A-Us constructs, we studied PKA, PKG, and ERK2 phosphorylation. This established that PKG and ERK2 do not clearly phosphorylate the N2A-Us (Supplementary Figure S2). In contrast, PKA did phosphorylate the N2A element, and its phosphorylation level was found to vary with the number of N2A-Us elements (Figures 5A,C). Consistent with this finding, mass-spectrometry analysis revealed a PKA phospho-site [Ser-9895 (NP_001254479.2), the same site as reported by Adams et al. (2019)], located at the C-terminal end of the N2A-Us and start of I81; see Figure 1D and Supplementary Figure S1. This site is identical in a wide range of species but in mouse, rat, and rabbit is a threonine (Supplementary Figure S3).

We also studied the effect of CARP on N2A phosphorylation by incubating the 2 N2A-Us protein with only PKA or PKA and CARP. This revealed that phosphorylation on the 2 N2A-Us protein was effectively blocked by CARP (Figures 5B,C, right 3 bars). Because CARP itself is not phosphorylated (Figure 5B), it is unlikely that CARP competes with N2A-Us, but, instead, CARP is likely to physically block PKA from interacting with the N2A-Us.

The effects of PKA phosphorylation of S9895 on the Lp of N2A-Us and Ig unfolding forces were also studied. No significant effects were detected on the Lp of the N2A-Us (Figure 5D, left two bars) nor on the Ig unfolding force (Figure 5D, right four bar pairs). This is not surprising considering that the location of the phosphorylation site is in/near the linker sequence between N2A-Us and I81 (see also section “Discussion”). In summary, PKA phosphorylates the N2A element, but this does not affect the mechanical properties of either the N2A-Us or the Ig unfolding forces.

**FIGURE 6:**
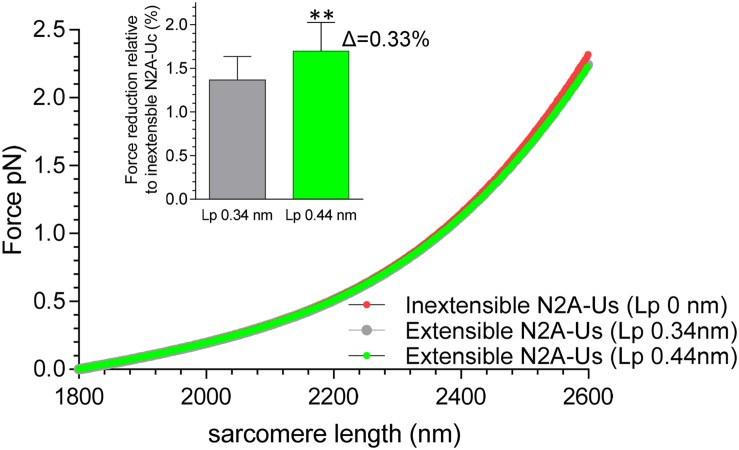
Effect of extensible N2A-Us on simulated single-molecule force-sarcomere length relation. A serially linked wormlike chain entropic spring model was used (see section “Materials and Methods”). The Lc of the N2A-Us was assumed to be 39 nm and the Lp either zero (i.e., inextensible, red symbols), 0.34 nm (no CARP, gray symbol), or 0.44 nm (CARP, green). The symbols largely overlap. The extensible N2A-Us lowers passive force slightly, on average within the shown sarcomere length range 1.7 ± 0.3% (Lp 0.44 nm) and 1.4 ± 0.3% (Lp 0.34 nm). Thus, increasing Lp from 0.34 to 0.44 (CARP effect) lowers force by an additional 0.33%. **p < 0.01.

Finally, the single-molecule force-sarcomere length relation of N2BA cardiac titin was simulated, in which the extensibility of the N2A-Us was taken into account (previously, only the tandem segments, the N2B element, and the PEVK were considered). Results show that inclusion of the N2A-Us modestly lowers passive force (within the shown sarcomere length range by 1.4%) and that including the effect of CARP (which increased Lp of N2A-Us from 0.34 to 0.44 nm) slightly increased this value (from 1.4 to 1.7%); see Figure 6.

## Discussion

It is well-established that in the I-band region of the sarcomere, titin functions as a molecular spring composed of distinct spring elements: the tandem Ig segments, the N2B element (N2B-Us), and the PEVK. The N2A element is located near the middle of the molecular spring, and the unique sequence that it contains (N2A-Us) is typically also viewed as a spring element. Additionally, CARP binds to both the N2A-Us and the flanking Ig domain I81, and CARP had been proposed to function analogously to a “clamp” between the N2A-Us and I81 and to reduce thereby the extensibility of the N2A-Us. The present study reveals that the N2A-Us behaves as a WLC with an Lc of 39 nm and an Lp of ∼0.35 nm and that CARP increases both the Lp of the N2A-Us and the unfolding force of I81. Furthermore, the N2A element is phosphorylated by PKA, phosphorylation is blocked by CARP, and phosphorylation does not alter the mechanical properties of the N2A element. Below we discuss these findings in detail.

### Protein Constructs

In order to obtain AFM results that can be easily interpreted, different protein constructs were used that all consist of four immunoglobulin (Ig) domains but a variable number of N2A-Us domains. This design allowed us to better identify the molecular origin of features in the force-extension curve. The power of this can be clearly seen in Figure 3, where the displacement of the curves obtained with the different protein constructs provides high confidence that the extension of the N2A-Us is 39 nm. If only the 1 N2A-Us molecule had been studied, this would not have been possible. Sequential unfolding of the Ig domains during extension of the constructs provided a characteristic single-molecule fingerprint, ensuring that the results were from single-molecules. To achieve specific attachment at the ends of the constructs, thiol chemistry was used to attach the N-terminal double cysteine residues to a gold-coated cantilever, and this was combined with a Halo-tag at the C-terminus to form an ester bond with a chloroalkane-functionalized glass side. Covalent tethering was confirmed by five force peaks in the force-extension curve, reflecting the unfolding of five protein domains contained in the constructs (four Ig domains and the Halo tag). The constructs were engineered such that the direction of force was applied through the Halo-tag N-terminus, since it is known that this results in a higher Halo-tag unfolding force (F_unfold_) than when force is applied through its C-terminus (Popa et al., 2013). As a result, the Halo-tag unfolds at a much higher force than the Ig domains (∼310 pN vs. ∼160 pN) and will therefore typically unfold last during full extension of the molecule. In conclusion, the constructs that were made ensured that single-molecules were studied and that force-extension curves were obtained from the full-length molecule. Importantly, by comparing results from constructs with 0, 1, and 2 N2A-Us domains, the properties of N2A-Us and Ig domain unfolding could be “dissected.”

### I80 and I81 Unfolding Force (F_unfold_)

The I80 and I81 F_unfold_ levels ranged from ∼125 pN (first force peak) to ∼165 pN (fourth force peak). The increase in F_unfold_ with peak number is a common feature of polyproteins (Carrion-Vazquez et al., 1999; Zhu et al., 2009) and can be explained by the stochastic nature of unfolding and the reduced likelihood of unfolding of a single domain in a chain with a lower number of remaining folded domains (Anderson et al., 2013). The F_unfold_ – peak number relation might also, in part, be due to a difference in the F_unfold_ between I80 and I81, although the difference is unlikely to be large, considering the gradual and linear increase of F_unfold_ with peak number (Figure 4C). Distinct F_unfold_ levels are seen when domains of different mechanical stability are linked (Li and Fernandez, 2003). The present study was conducted at a pulling speed of 400 nm/s, and comparing the results to earlier studies in which Ig domains were stretched at the same or a similar speed shows that I80 and I81 unfold at lower force levels than domains from the distal and middle tandem Ig segments (Carrion-Vazquez et al., 1999; Li et al., 2002; Watanabe et al., 2002a; Zhu et al., 2009) but at levels similar or slightly higher than domains from the proximal tandem Ig segment (Li and Fernandez, 2003; Anderson et al., 2013). For example, the proximal tandem Ig segment domain I1 unfolds at ∼110 pN (pulling speed 400 nm/sec) (Li and Fernandez, 2003), and I10 unfolds at ∼120 pN (pulling speed 1000 nm/s) (Anderson et al., 2013). Interestingly, the flanking Ig domains of the N2B-Us (I24 and I25) unfold at 125–165 pN (pulling speed 500 nm/s) (Zhu et al., 2009), which is nearly identical to the I80 and I81 F_unfold_. Although it is uncertain to what extent unfolding of Ig domains occurs under physiological conditions (Trombitas et al., 1998b; Chen et al., 2015), if it does occur, this might involve Ig domains from the proximal tandem Ig segment and domains that flank the unique sequences of both the N2B and N2A elements.

### N2A-Us Extension

The low-force phase of the extension curve prior to the first unfolding peak can be fit well with the WLC force equation, and comparing the thus-obtained contour length (Lc) of the constructs with 0,1, and 2 N2A-Us domains convincingly shows that a single N2A-Us domain has an Lc of ∼39 nm. Considering that the N2A-Us contains 104 residues, this Lc value indicates an average residue spacing of 3.75 Å. This value is close to the 3.8 Å maximal residue spacing of an unfolded polypeptide chain (Cantor, 1980), suggesting that no secondary structures exist in the N2A-Us when the first Ig domain unfolds. However, a recent structural study revealed that N2A-Us has α-helical-rich secondary structures (∼60–70% helical content), with helices arranged into a 3D-fold, similar to the MyBP-C tri-helix bundle (Howarth et al., 2012; Bezold et al., 2013; Zhou et al., 2016). The axial residue spacing along the α-helix axis is 1.47 Å (Pauling et al., 1951), and assuming that 70% of the N2A-Us is α-helical results in an Lc of 10.7 nm (the α-helical region will be more compact than this if the α-helices adopt 3-D folds). Considering the much longer Lc value that was obtained in this study, α-helices must have largely unfolded well before the first Ig domain unfolding peak was registered. No force peaks were observed in the traces leading up to the first Ig domain unfolding peak, either in the 1 N2A-Us or 2 N2A-Us construct. In a previous AFM study, we showed that when pulling the α-helical protein spectrin, clear unfolding peaks are detectable in the 15–25 pN force regime (Watanabe et al., 2002a), and we are confident that if similar force peaks had occurred while pulling N2A-Uc, they would also have been detected.

The lack of unfolding peaks during N2A-Us extension suggests either that the unfolding force of the α-helical secondary structures is below the resolution limit of our AFM (∼10 pN) or that unfolding occurs non-cooperatively and does not produce a distinct force peak. The lack of force peaks due to N2A-Us unfolding is consistent with AFM measurements on α-helical domains from myosin-10 in which unfolding peaks were also absent (Wolny et al., 2014). This myosin-10 study included molecular dynamics (MD) simulations that showed gradual unfolding of α-helical domains at an approximately constant force. This force varied with pulling speed and was ∼50 pN at the slowest pulling speed used in the MD simulation, 1 m/s. Considering that the present AFM study pulled N2A-Us 2.5 × 10^4^ slower than the MD simulation (400 nm/s) and the well-known speed-dependence of unfolding, the force level at which α-helical domains unfold might be quite low at our experimental pulling speed and thus might be undetectable. Future studies with higher force resolution techniques (e.g., laser tweezers) will be required to establish how the N2A-Us extends at low force levels. Based on the present AFM study, the N2A-Us can be modeled as a WLC with Lc 39 nm and Lp 0.34 nm.

### Role of N2A-Us in the Passive Force-Sarcomere Length Relation of Titin

To evaluate the role that the N2A-Us plays in passive force generation, the molecular spring region of cardiac N2BA titin was simulated as a serially linked WLC comprised of tandem Ig segments, the N2B-Uc, the PEVK, and the N2A-Us. The Lp and Lc values for the first three spring elements were based on previous work (see section “Materials and Methods”) and for the N2A-Us on the present study. The simulated force-sarcomere length relation (Figure 6) shows that, within the evaluated sarcomere length range, the compliance provided by the N2A-Us only slightly lowers passive force, on average by 1.4%. This effect is expected to be even smaller in fetal cardiac titin and skeletal muscle titin because their PEVK and tandem Ig segments are longer than in adult cardiac N2BA titin (Freiburg et al., 2000; Lahmers et al., 2004), and the small effect of N2A-Us compliance on passive force will be further “diluted.” The effect is also expected to be less if α-helical structures exist in the N2A-Us that gradually unfold (see above). Thus, the extensibility of the N2A-Us has a negligible effect on the level of passive force that titin generates, and, in contrast to the tandem Ig segments, the N2B-Us, and the PEVK, the N2A-Us is unlikely to function primarily as a molecular spring element.

### CARP

The presence of CARP slightly increased both the Lp of the N2A-Us and the unfolding force (F_unfold_) of its flanking Ig domains (Figure 4), findings consistent with earlier biochemical studies that showed that CARP interacts with both the N2A-Us and I81 (Zhou et al., 2016). Based on this dual interaction, it has been proposed that CARP regulates the stretch response of the N2A element (Zhou et al., 2016). However, the predicted effect of the increase in Lp on passive force is, at 0.4%, miniscule (Figure 6), and it seems unlikely that this is an important biological function of CARP binding to titin. The increased F_unfold_ could be relevant if I81 is at risk of unfolding, which might be the case considering that the measured F_unfold_ is comparatively low (see above) and CARP is upregulated under conditions of mechanical stress where unfolding is more likely to occur (Barash et al., 2004; van der Pijl et al., 2019). At present, it cannot be excluded that, *in vivo*, CARP does affect passive force, since multiple complexities occur in the sarcomere that are absent in single-molecule assays (if the sarcomere thin filaments are present, CARP might dimerize and crosslink different filaments, etc.) The increased F_unfold_ that our single-molecule study revealed (Figure 4C) suggests that it is possible that CARP functions as a chaperone. This would be similar to αB-crystallin, which acts as a chaperone for the N2B element by increasing the F_unfold_ of the Ig domains that flank the N2B-Us (Zhu et al., 2009), and αB-crystallin is also upregulated under conditions of stress (Bullard et al., 2004). Thus, CARP might function as a molecular chaperone that protects I81 from unfolding when mechanical stress is high.

### Phosphorylation and Signaling

The N2A-Us was found to contain a PKA site (S9895 in NP_001254479.2) at the border between the N2A-Us and I81, outside the α-helical N2A-Us region that was discussed above. This site is the same as the PKA site reported by Adams et al. (2019) and presumably also the same site that was phosphorylated by PKA in the study Lun et al. (2014) (the authors did not locate the amino acid(s) that was(were) phosphorylated by PKA). Interestingly the N2A element has been reported to be a PKG substrate and not a PKA substrate by Kruger et al. (2009). The same laboratory showed later that the PKG phosphosite is S9895 (Hisman et al., 2011), i.e., the same site as the PKA site that we and Adams et al. (2019) found (note that the reported PKG site S8651 used NP_596869.4 as the reference sequence and that this site is the same as S9895 in NP_001254479.2 that we used). The reason for the discrepancy is not clear. All phosphorylation studies used the human N2A sequence, and a species difference is therefore ruled out. Considering the convincing phosphorylation of N2A-Us by PKA that we found (Figure 5A) and the clear correspondence between our study and the studies from two other laboratories (Lun et al., 2014; Adams et al., 2019) the case for N2A-Us as a PKA substrate with as phospho-site SS9895 is strong.

No effect of phosphorylation was found on the persistence length of the N2A-Us nor on the unfolding force of I81. This is consistent with the location of the PKA site in what is likely an unstructured region that links the N2A-Us to I81. Thus unlike PKA phosphorylation of the N2B-Us, which increases the compliance of the N2B-Us (Yamasaki et al., 2002; Kruger et al., 2009), phosphorylation of Ser-9895 does not affect the mechanical properties of N2A-Us. Considering that the N2A-Us PKA site is well conserved (Supplementary Figure S3), it is likely that PKA phosphorylation does have a biological function. This might be regulating the binding affinity between the N2A-Us and other proteins. For example, CARP binding could be sensitive to PKA phosphorylation. However, despite CARP binding precluding N2A-Us phosphorylation (Figures 5B,C), N2A phosphorylation does not abolish the binding of CARP (results not shown). CARP is a member of a conserved gene family referred to as MARPs (muscle ankyrin repeat proteins) that also includes ankrd-2/Arpp/MARP2 and DARP/MARP3. All MARP family members contain within their ankyrin repeat region a binding site for the N2A element of titin (Miller et al., 2003), and thus it is possible that unlike CARP, other MARP family members do bind to titin in a manner that depends on S9895 phosphorylation. Another N2A-Us-binding protein with an affinity that might be controlled through S9895 phosphorylation is the lysine methyltransferase Smyd2. Smyd2 has been shown to bind to N2A-Us and to play an important role in the structural stabilization of sarcomeric proteins, including protecting the N2A element from degradation (Voelkel et al., 2013). A final possible candidate with titin-binding affinity that might be controlled by PKA phosphorylation of S9895 is calpain3. Calpain3 has multiple binding sites to titin, including the I80-N2A-Us region (Hayashi et al., 2008), and binding suppresses the activity of this protease (Ono et al., 2006). *In vitro* cell culture experiments have shown that the binding of calpain3 is stretch-dependent and that increasing sarcomere length increases calpain3 accumulation at the N2A site (Ojima et al., 2010). Clearly, additional studies are required to establish the functional role of PKA phosphorylation of S9895 and whether this includes regulating the binding affinity and function of titin-binding proteins.

## Conclusion

In AFM experiments, the N2A-Us behaves as an entropic spring with an Lp of ∼0.35 nm and an Lc of 39 nm. CARP increases the unfolding force of I81 and might thereby act as a molecular chaperone that protects I81 from unfolding when mechanical stress is high. The N2A-Us is a PKA substrate, and phosphorylation can be blocked by CARP. Simulating the force–sarcomere length relation of a single titin molecule containing all spring elements shows that the compliance of the N2A-Us only slightly reduces passive force, suggesting that it is unlikely that the N2A element has a mechanical function *per se*. Considering that the N2A element is an anchoring hub for signaling proteins, it is likely that the compliance of the N2A-Us has local effects on binding of signaling molecules and that it contributes thereby to strain- and phosphorylation-dependent mechano-signaling.

## Data Availability Statement

The datasets generated for this study can be found in the Mass Spectrometry data for this study can be found at the Harvard Dataverse (https://doi.org/10.7910/DVN/OZQD7G).

## Author Contributions

All authors listed have made a substantial, direct and intellectual contribution to the work, and approved it for publication. VK made contributions to the acquisition and analysis of data for this work and drafting of the revised manuscript, provided final approval of the version to be published, and agreed to be accountable for all aspects of the work in ensuring that questions related to the accuracy or integrity of any part of the work are appropriately investigated and resolved.

## Conflict of Interest

The authors declare that the research was conducted in the absence of any commercial or financial relationships that could be construed as a potential conflict of interest.

## Footnotes

1 https://doi.org/10.7910/DVN/OZQD7G
