# Supplementary material for: Single-Molecule Force Spectroscopy on the N2A Element of Titin: Effects of Phosphorylation and CARP
Source: Front Physiol. 2020 Mar 18;11:173. doi: 10.3389/fphys.2020.00173 (PMC7093598; doi:10.3389/fphys.2020.00173)
Supplement: Supplementary file 1 [file Presentation_1.pptx]

## Slide 1
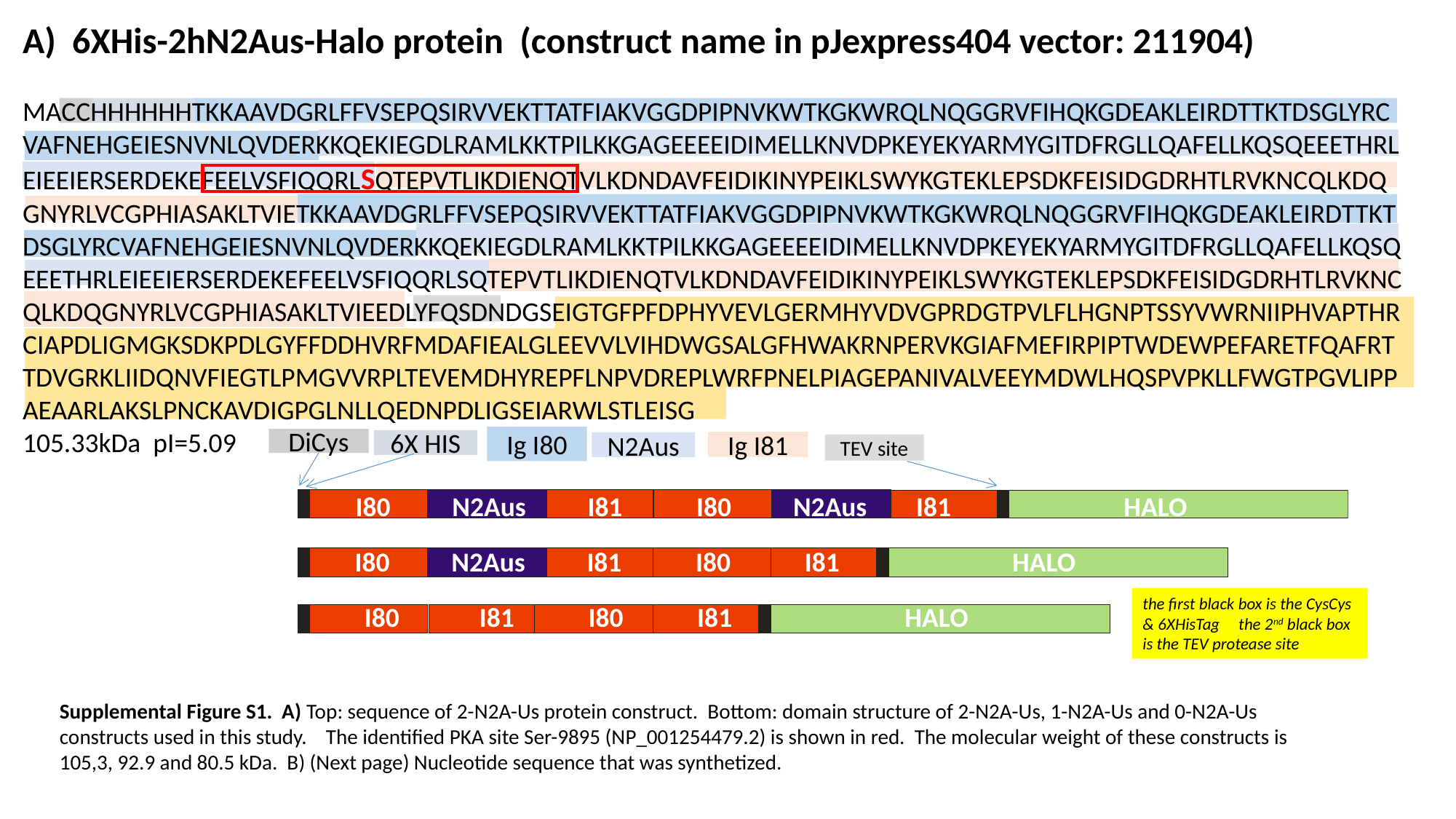

A) 6XHis-2hN2Aus-Halo protein (construct name in pJexpress404 vector: 211904)
MACCHHHHHHTKKAAVDGRLFFVSEPQSIRVVEKTTATFIAKVGGDPIPNVKWTKGKWRQLNQGGRVFIHQKGDEAKLEIRDTTKTDSGLYRCVAFNEHGEIESNVNLQVDERKKQEKIEGDLRAMLKKTPILKKGAGEEEEIDIMELLKNVDPKEYEKYARMYGITDFRGLLQAFELLKQSQEEETHRLEIEEIERSERDEKEFEELVSFIQQRLSQTEPVTLIKDIENQTVLKDNDAVFEIDIKINYPEIKLSWYKGTEKLEPSDKFEISIDGDRHTLRVKNCQLKDQGNYRLVCGPHIASAKLTVIETKKAAVDGRLFFVSEPQSIRVVEKTTATFIAKVGGDPIPNVKWTKGKWRQLNQGGRVFIHQKGDEAKLEIRDTTKTDSGLYRCVAFNEHGEIESNVNLQVDERKKQEKIEGDLRAMLKKTPILKKGAGEEEEIDIMELLKNVDPKEYEKYARMYGITDFRGLLQAFELLKQSQEEETHRLEIEEIERSERDEKEFEELVSFIQQRLSQTEPVTLIKDIENQTVLKDNDAVFEIDIKINYPEIKLSWYKGTEKLEPSDKFEISIDGDRHTLRVKNCQLKDQGNYRLVCGPHIASAKLTVIEEDLYFQSDNDGSEIGTGFPFDPHYVEVLGERMHYVDVGPRDGTPVLFLHGNPTSSYVWRNIIPHVAPTHRCIAPDLIGMGKSDKPDLGYFFDDHVRFMDAFIEALGLEEVVLVIHDWGSALGFHWAKRNPERVKGIAFMEFIRPIPTWDEWPEFARETFQAFRTTDVGRKLIIDQNVFIEGTLPMGVVRPLTEVEMDHYREPFLNPVDREPLWRFPNELPIAGEPANIVALVEEYMDWLHQSPVPKLLFWGTPGVLIPPAEAARLAKSLPNCKAVDIGPGLNLLQEDNPDLIGSEIARWLSTLEISG
105.33kDa pI=5.09
Ig I80
DiCys
6X HIS
Ig I81
N2Aus
TEV site
I80 N2Aus I81 I80 N2Aus I81 HALO
I80 N2Aus I81 I80 I81 HALO
I80 I81 I80 I81 HALO
the first black box is the CysCys & 6XHisTag the 2nd black box is the TEV protease site
Supplemental Figure S1. A) Top: sequence of 2-N2A-Us protein construct. Bottom: domain structure of 2-N2A-Us, 1-N2A-Us and 0-N2A-Us constructs used in this study. The identified PKA site Ser-9895 (NP_001254479.2) is shown in red. The molecular weight of these constructs is 105,3, 92.9 and 80.5 kDa. B) (Next page) Nucleotide sequence that was synthetized.

## Slide 2
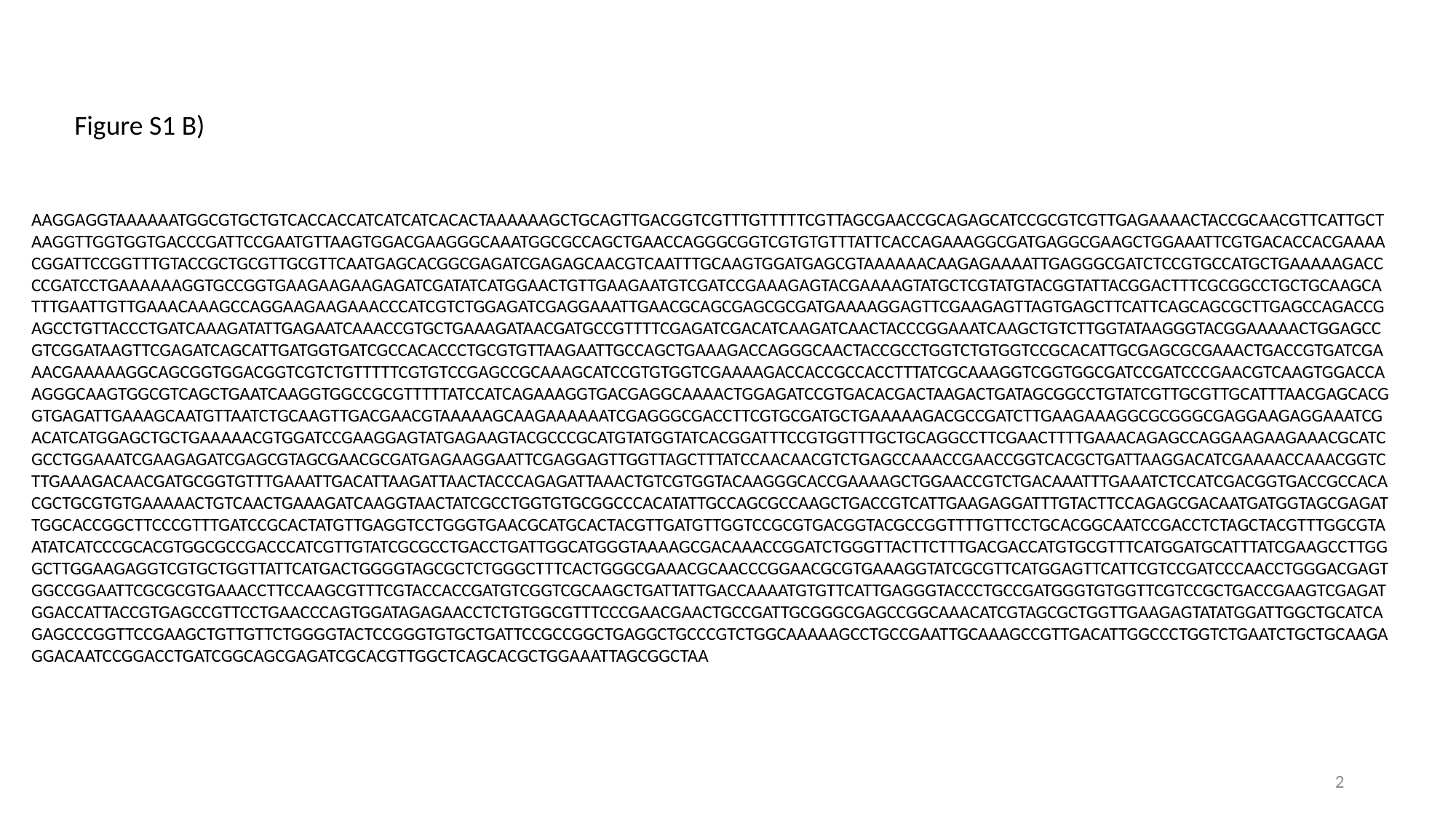

Figure S1 B)
AAGGAGGTAAAAAATGGCGTGCTGTCACCACCATCATCATCACACTAAAAAAGCTGCAGTTGACGGTCGTTTGTTTTTCGTTAGCGAACCGCAGAGCATCCGCGTCGTTGAGAAAACTACCGCAACGTTCATTGCTAAGGTTGGTGGTGACCCGATTCCGAATGTTAAGTGGACGAAGGGCAAATGGCGCCAGCTGAACCAGGGCGGTCGTGTGTTTATTCACCAGAAAGGCGATGAGGCGAAGCTGGAAATTCGTGACACCACGAAAACGGATTCCGGTTTGTACCGCTGCGTTGCGTTCAATGAGCACGGCGAGATCGAGAGCAACGTCAATTTGCAAGTGGATGAGCGTAAAAAACAAGAGAAAATTGAGGGCGATCTCCGTGCCATGCTGAAAAAGACCCCGATCCTGAAAAAAGGTGCCGGTGAAGAAGAAGAGATCGATATCATGGAACTGTTGAAGAATGTCGATCCGAAAGAGTACGAAAAGTATGCTCGTATGTACGGTATTACGGACTTTCGCGGCCTGCTGCAAGCATTTGAATTGTTGAAACAAAGCCAGGAAGAAGAAACCCATCGTCTGGAGATCGAGGAAATTGAACGCAGCGAGCGCGATGAAAAGGAGTTCGAAGAGTTAGTGAGCTTCATTCAGCAGCGCTTGAGCCAGACCGAGCCTGTTACCCTGATCAAAGATATTGAGAATCAAACCGTGCTGAAAGATAACGATGCCGTTTTCGAGATCGACATCAAGATCAACTACCCGGAAATCAAGCTGTCTTGGTATAAGGGTACGGAAAAACTGGAGCCGTCGGATAAGTTCGAGATCAGCATTGATGGTGATCGCCACACCCTGCGTGTTAAGAATTGCCAGCTGAAAGACCAGGGCAACTACCGCCTGGTCTGTGGTCCGCACATTGCGAGCGCGAAACTGACCGTGATCGAAACGAAAAAGGCAGCGGTGGACGGTCGTCTGTTTTTCGTGTCCGAGCCGCAAAGCATCCGTGTGGTCGAAAAGACCACCGCCACCTTTATCGCAAAGGTCGGTGGCGATCCGATCCCGAACGTCAAGTGGACCAAGGGCAAGTGGCGTCAGCTGAATCAAGGTGGCCGCGTTTTTATCCATCAGAAAGGTGACGAGGCAAAACTGGAGATCCGTGACACGACTAAGACTGATAGCGGCCTGTATCGTTGCGTTGCATTTAACGAGCACGGTGAGATTGAAAGCAATGTTAATCTGCAAGTTGACGAACGTAAAAAGCAAGAAAAAATCGAGGGCGACCTTCGTGCGATGCTGAAAAAGACGCCGATCTTGAAGAAAGGCGCGGGCGAGGAAGAGGAAATCGACATCATGGAGCTGCTGAAAAACGTGGATCCGAAGGAGTATGAGAAGTACGCCCGCATGTATGGTATCACGGATTTCCGTGGTTTGCTGCAGGCCTTCGAACTTTTGAAACAGAGCCAGGAAGAAGAAACGCATCGCCTGGAAATCGAAGAGATCGAGCGTAGCGAACGCGATGAGAAGGAATTCGAGGAGTTGGTTAGCTTTATCCAACAACGTCTGAGCCAAACCGAACCGGTCACGCTGATTAAGGACATCGAAAACCAAACGGTCTTGAAAGACAACGATGCGGTGTTTGAAATTGACATTAAGATTAACTACCCAGAGATTAAACTGTCGTGGTACAAGGGCACCGAAAAGCTGGAACCGTCTGACAAATTTGAAATCTCCATCGACGGTGACCGCCACACGCTGCGTGTGAAAAACTGTCAACTGAAAGATCAAGGTAACTATCGCCTGGTGTGCGGCCCACATATTGCCAGCGCCAAGCTGACCGTCATTGAAGAGGATTTGTACTTCCAGAGCGACAATGATGGTAGCGAGATTGGCACCGGCTTCCCGTTTGATCCGCACTATGTTGAGGTCCTGGGTGAACGCATGCACTACGTTGATGTTGGTCCGCGTGACGGTACGCCGGTTTTGTTCCTGCACGGCAATCCGACCTCTAGCTACGTTTGGCGTAATATCATCCCGCACGTGGCGCCGACCCATCGTTGTATCGCGCCTGACCTGATTGGCATGGGTAAAAGCGACAAACCGGATCTGGGTTACTTCTTTGACGACCATGTGCGTTTCATGGATGCATTTATCGAAGCCTTGGGCTTGGAAGAGGTCGTGCTGGTTATTCATGACTGGGGTAGCGCTCTGGGCTTTCACTGGGCGAAACGCAACCCGGAACGCGTGAAAGGTATCGCGTTCATGGAGTTCATTCGTCCGATCCCAACCTGGGACGAGTGGCCGGAATTCGCGCGTGAAACCTTCCAAGCGTTTCGTACCACCGATGTCGGTCGCAAGCTGATTATTGACCAAAATGTGTTCATTGAGGGTACCCTGCCGATGGGTGTGGTTCGTCCGCTGACCGAAGTCGAGATGGACCATTACCGTGAGCCGTTCCTGAACCCAGTGGATAGAGAACCTCTGTGGCGTTTCCCGAACGAACTGCCGATTGCGGGCGAGCCGGCAAACATCGTAGCGCTGGTTGAAGAGTATATGGATTGGCTGCATCAGAGCCCGGTTCCGAAGCTGTTGTTCTGGGGTACTCCGGGTGTGCTGATTCCGCCGGCTGAGGCTGCCCGTCTGGCAAAAAGCCTGCCGAATTGCAAAGCCGTTGACATTGGCCCTGGTCTGAATCTGCTGCAAGAGGACAATCCGGACCTGATCGGCAGCGAGATCGCACGTTGGCTCAGCACGCTGGAAATTAGCGGCTAA
2

## Slide 3
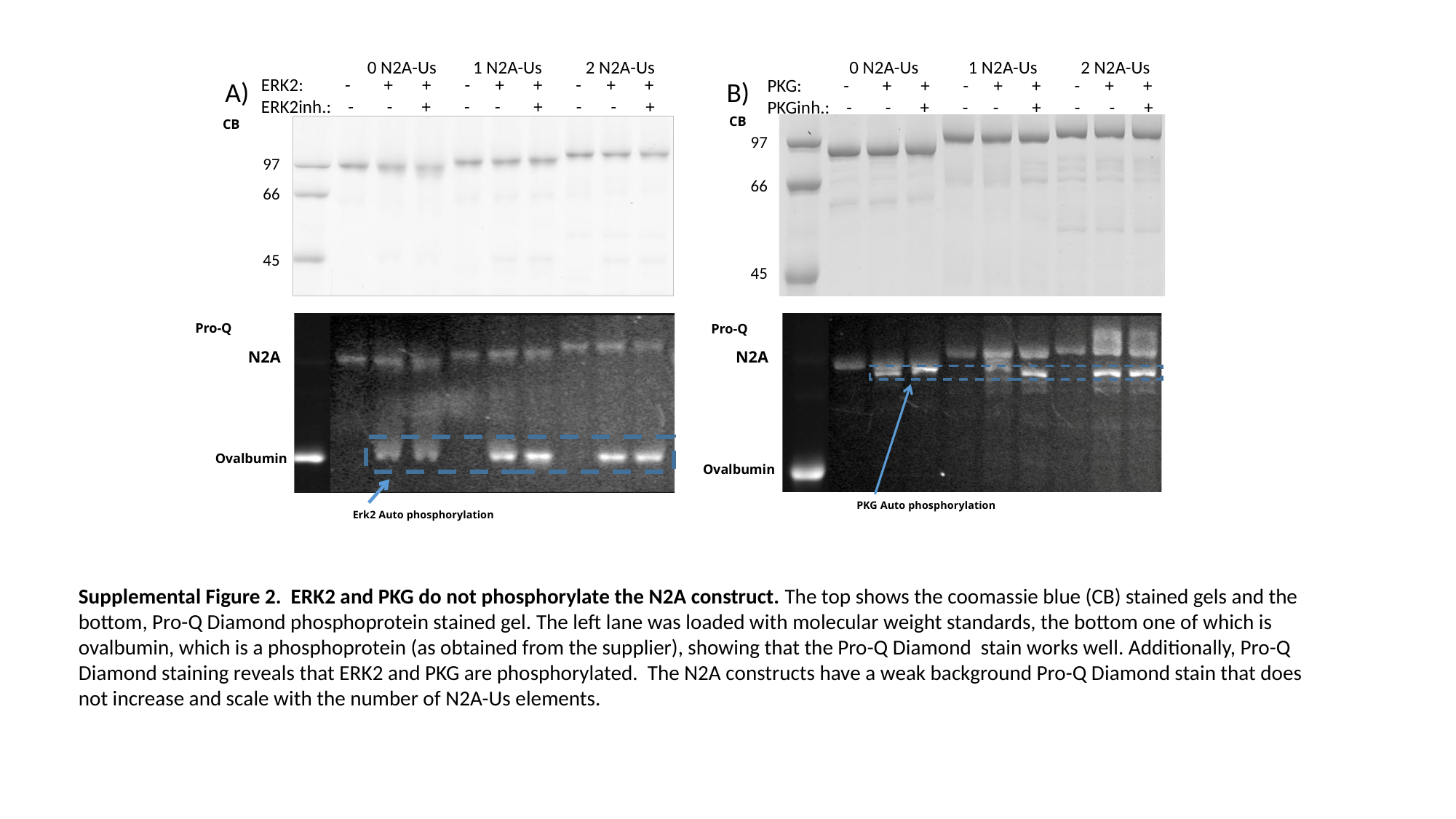

0 N2A-Us
1 N2A-Us
2 N2A-Us
0 N2A-Us
1 N2A-Us
2 N2A-Us
ERK2: - + + - + + - + +
ERK2inh.: - - + - - + - - +
PKG: - + + - + + - + +
PKGinh.: - - + - - + - - +
CB
97
66
45
97
66
45
CB
Pro-Q
Pro-Q
N2A
N2A
Ovalbumin
Ovalbumin
PKG Auto phosphorylation
Erk2 Auto phosphorylation
A)
B)
Supplemental Figure 2. ERK2 and PKG do not phosphorylate the N2A construct. The top shows the coomassie blue (CB) stained gels and the bottom, Pro-Q Diamond phosphoprotein stained gel. The left lane was loaded with molecular weight standards, the bottom one of which is ovalbumin, which is a phosphoprotein (as obtained from the supplier), showing that the Pro-Q Diamond stain works well. Additionally, Pro-Q Diamond staining reveals that ERK2 and PKG are phosphorylated. The N2A constructs have a weak background Pro-Q Diamond stain that does not increase and scale with the number of N2A-Us elements.
Figure 6. PKA phosphorylation assay on 0, 1, and 2 N2A-Uc N2A elements. A) Top: Sypro Ruby stained gel samples derived from proteins that had been either not PKA treated, PKA treated, or PKA treated in the presence of a PKA inhibitor. Lane 1 is a molecular mass ladder (the 45 kDa protein is ovalbumin, which is phosphorylated) The samples revealed bands near ~97 kDa representing the N2A-Uc proteins. The band at 45 kDa is derived from PKA. Bottom: Pro-Q diamond stained gel shows no phosphorylation in the PKA-treated 0 N2A-Uc protein, a clear phosphorylation signal in 1 N2A-Uc protein and an even stronger signal in the 2 N2A-Uc protein. Phosphorylation is abolished by the PKA inhibitor. B) Quantitative analysis shows that PKA phosphorylation requires the presence of the N2A-Uc. C) Primary sequence of Ig80(red)/N2A-Uc(green)/Ig81(red) fragment with indicated in bold the serine residue at the end of the N2A-Uc and threonine (T) and at the start of the Ig81 that were identified by Mass Spec as PKA substrates.

## Slide 4
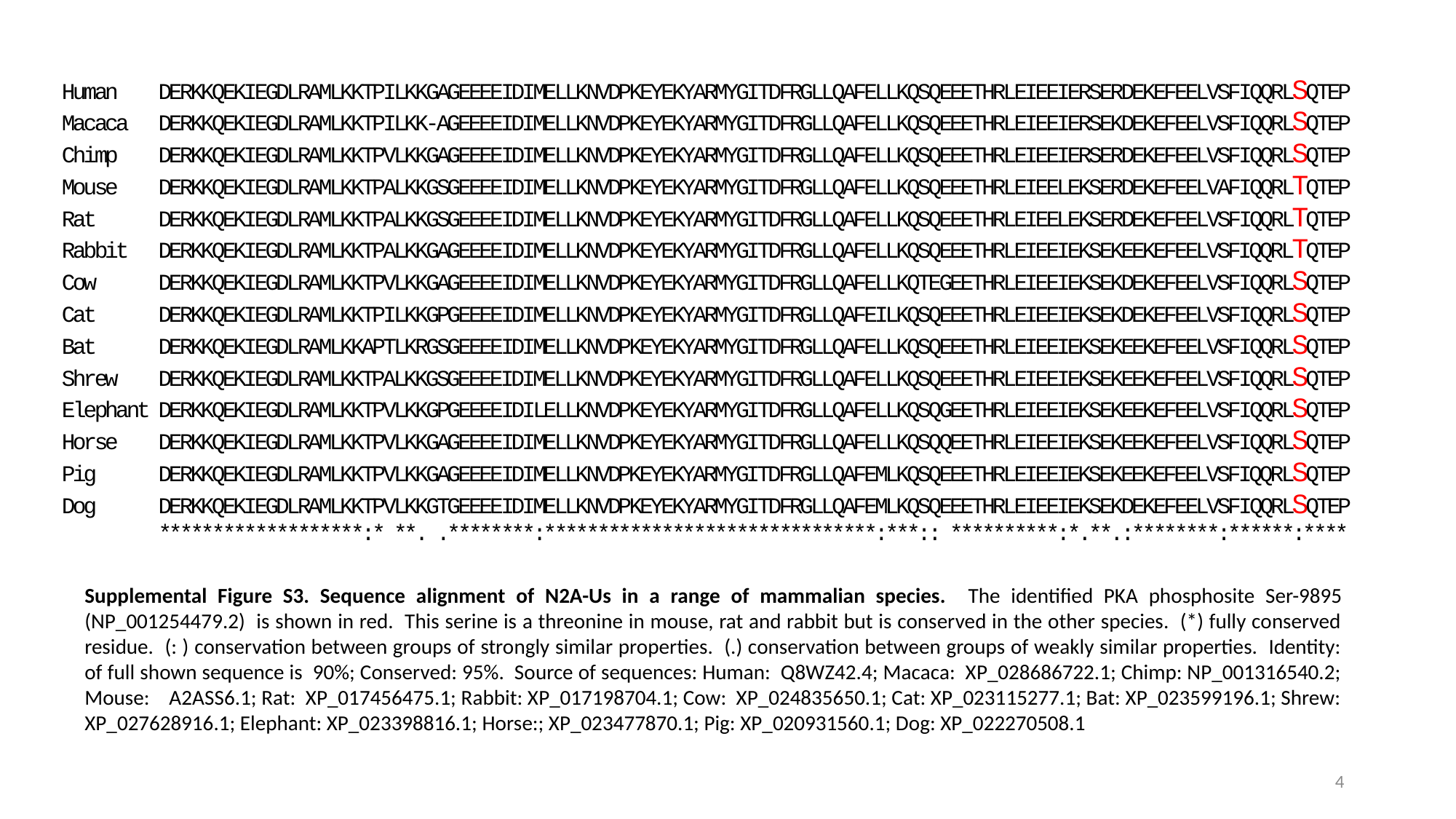

Supplemental Figure S3. Sequence alignment of N2A-Us in a range of mammalian species. The identified PKA phosphosite Ser-9895 (NP_001254479.2) is shown in red. This serine is a threonine in mouse, rat and rabbit but is conserved in the other species. (*) fully conserved residue. (: ) conservation between groups of strongly similar properties. (.) conservation between groups of weakly similar properties. Identity: of full shown sequence is 90%; Conserved: 95%. Source of sequences: Human: Q8WZ42.4; Macaca: XP_028686722.1; Chimp: NP_001316540.2; Mouse: A2ASS6.1; Rat: XP_017456475.1; Rabbit: XP_017198704.1; Cow: XP_024835650.1; Cat: XP_023115277.1; Bat: XP_023599196.1; Shrew: XP_027628916.1; Elephant: XP_023398816.1; Horse:; XP_023477870.1; Pig: XP_020931560.1; Dog: XP_022270508.1
4
